# The Relationship Between Locus of Control and Religious Behavior and Beliefs in a Large Population of Parents: An Observational Study

**DOI:** 10.3389/fpsyg.2020.01462

**Published:** 2020-06-25

**Authors:** Yasmin Iles-Caven, Steven Gregory, Genette Ellis, Jean Golding, Stephen Nowicki

**Affiliations:** ^1^Centre for Academic Child Health, Population Health Sciences, Bristol Medical School, University of Bristol, Bristol, United Kingdom; ^2^Department of Psychology, Emory University, Atlanta, GA, United States

**Keywords:** ALSPAC, longitudinal cohort, locus of control, Rotter, religiosity, behavior, belief

## Abstract

The purpose of the present study was to examine, in a large representative population, the association between self-reported religious beliefs, attitudes and behavior and locus of control (LOC) of reinforcement as defined by Rotter. Results of previous research have failed to clearly determine what, if any, associations existed. In this study, analyses showed individuals with an internal LOC were not only more likely to believe in a divine power, to admit a divine power helped them in the past, to ask for help from a divine power in the future, to attend places of worship more often than those who were external, but also that they were significantly more likely to maintain their religious beliefs and behaviors over a 6 year period compared to those with an external orientation. Additional exploratory analyses by gender revealed that compared to internal men, internal women were significantly more involved in all indicators of religious belief and action except for attending church weekly and obtaining help from religious leaders where internal men were higher. The present findings support the association between the generalized expectancy of LOC as defined by Rotter and religious beliefs, attitudes and behaviors.

## Introduction

Considerable research has focused on religious attitudes, beliefs and behavior and the factors that may impact on them (see Koenig et al., 2012). This focus has recently become more intense in western countries because of data suggesting decreasing interest in religious belief and activity. In the United Kingdom, western Europe and the United States for example, surveys have shown that the general population is becoming more secular (Office for National Statistics, 2012; Pew Forum on Religion and Public Life, 2014; Chaves, 2017). Twenge and colleagues (Twenge et al., 2015) examined data on 11.2 million American adolescents (1966–2014) and found that those surveyed in the 2010s were significantly less religious that in previous generations. Their suggestion, that there is a connection between less religious involvement and rising individualism (focusing on self rather than on others and society), may have some theoretical and logical basis: “…the primary cultural change in recent decades in the US is an increase in individualism… (p. 13).”

An individual difference characteristic that reflects the degree of individualism is called locus of control (LOC). According to Rotter (1966) who introduced the construct to the psychological community over half a century ago, the more individuals perceive connections between their behavior and outcomes the more “internal” they are in contrast to those who are more prone to view their outcomes as being determined by luck, fate, chance or powerful others who are called “external.” Results of thousands of studies using measures of LOC consistent with Rotter’s definition strongly suggest that internality is related to more positive outcomes overall than externality (Rotter, 1966, 1975; Lefcourt, 1976; Nowicki, 2016).

A question to be answered in the present study is whether a greater likelihood of engagement in religious activity and/or belief is related to being internally or externally controlled. There is a clear theoretical reason for considering LOC’s involvement with religious belief and action. In his social learning theory, Rotter defined LOC of reinforcement as a generalized “problem solving” expectancy that is especially “triggered” when individuals are trying to resolve serious problems; and since there is no greater problem than figuring out why we exist then individual differences in LOC may very well be related to differences in religious belief and activity.

However, researchers do not even agree on the direction of the association. As Coursey et al. (2013) put it: “The impact of religion on an individual’s sense of personal agency is ambiguous. Religious commitment often carries with it the acknowledgment of divine presence and power in one’s life. One manifestation of this belief implies a relinquishing of personal or internal control and an acceptance of God’s will or external control” (p. 348). Others though, have argued that God control and personal control can co-exist by forming a relationship with God that displays mutuality (Zahl and Gibson, 2008). Unfortunately results from past studies of religious belief and activities and LOC do not offer much help because they too have found a variety of associations. In some instances, externality (Wiley, 2006), internality (Wigert, 2002), or neither (Lowis et al., 2009) were related to religiosity.

The absence of clear and consistent findings regarding a religiosity/LOC association may have a number of contributors including participants differing dramatically in age, as well as researchers’ tendency to use a variety of measures of religiosity and LOC as though they were synonymous.

In terms of what is meant by religiosity, Coursey et al. (2013) suggested that intrinsic and extrinsic aspects of religiosity should be measured and studied separately because they are independent or orthogonal to one another. Intrinsic religiosity reflects a belief in the tenets of the accepted religion and behaviors consistent with those tenets. On the other hand, extrinsic religiosity refers to how much one uses religion as a means to an end to achieve non-religious goals such as social support, sense of community and the like. In many ways these indicators of religiosity are orthogonal to one another and should not be used interchangeably. Based on the results of a meta-analysis (Coursey et al., 2013), when religiosity is measured intrinsically, it is significantly associated with LOC internality when LOC is measured by Rotter’s scale.

Not only may the use of different measures of religiosity contribute to inconsistent findings regarding its association with LOC, but also there are potential negative consequences of researchers using a number of different tests to assess the LOC construct. Nowicki and Duke (2016) pointed out that this is a growing problem across all areas of psychology where the term “locus of control” is applied to literally hundreds of different tests without data to support appropriate use of the LOC label (Skinner, 1996): that is, assuming that because a test is called a LOC test it is measuring the LOC construct as defined by Rotter (1966). Kelley (1927) called this the “Jingle” fallacy; using different tests to measure the same construct without providing support that this is true. When studying LOC, Nowicki et al. (2018) went so far as to call for researchers to only use tests that provide sufficient construct validity evidence that they are measuring what Rotter defined as “locus of control of reinforcement.”

We have used the term “religiosity” as a catch-all for religious/spiritual beliefs and behaviors in this paper. Religion is a multi-dimensional construct (Koenig et al., 2014) and perhaps we are using the word “religiosity” inappropriately here.

The main aim of the present study therefore is to evaluate the association between LOC as measured by a test shown to be a valid measure of LOC consistent with Rotter’s definition and self-reported religious beliefs and activities of participants from a large representative population. It is predicted that individuals who are high in religiosity as measured both by attendance and belief will have a more internal LOC than those low in religiosity. This is based on the findings of Coursey et al. (2013) who found internality associated with intrinsic religiosity in a younger population when Rotter’s scale was used. When researchers used LOC tests constructed to be consistent with the construct as defined by Rotter, greater religiosity as measured by activity within the religion was found to be associated with greater internality. This is consistent with reasoning from Rotter’s social learning theory which suggests that compared to externals, internals are more likely to expect their efforts to produce solutions for whatever problem they are trying to solve, to gather more information, be more persistent and responsible, and more able to delay obtaining rewards. The present study not only used a construct validated LOC scale, developed to be consistent with Rotter’s development of the concept, but one which may be more appropriate for use in a representative population because of its easier reading level (Nowicki and Duke, 1974).

It has been reported that both religiosity/spirituality and LOC do influence health-related behaviors and outcomes, which is important for public health. Specific measures of LOC in various domains such as health, multi-dimensional health and spiritual health have been devised that attempt to predict health-related behaviors and outcomes. These scales may not be consistent with Rotter’s definition and are often used on small cross-sectional studies (see Timmins and Martin, 2019 for a review). To this end we attempt to explore the relationship between a general measure of LOC and religion/spirituality in the ALSPAC longitudinal cohort as a first step toward further analyses.

Our second aim was to examine how stable the religiosity and LOC association was over time. While Kay et al. (2010) reviewed the research concerned with the compensatory give and take of LOC depending on situational demands, no one to our knowledge has been able to assess whether internals or externals remain more stable in their religious beliefs or actions. Because past research has revealed internals to be more persistent over time because they see their actions affecting what happens to them, it is predicted that they will be more stable in their religious beliefs and actions than externals.

## Materials and Methods

### The Study Population

This paper uses data collected as part of the Avon Longitudinal Study of Parents and Children (ALSPAC). This pre-birth cohort was designed to determine environmental and genetic factors associated with the development, health and well-being of the resulting offspring (Golding and the Alspac Study Team, 2004; Boyd et al., 2013; Fraser et al., 2013). A major component of the study design was to obtain, from the parents, details of their own personalities, moods and attitudes, including measures of their LOC and religiosity, prior to the birth of the child.

ALSPAC recruited pregnant women who resided in Avon, in the south-west of the UK, with an expected date of delivery between 1st April 1991 and 31st December 1992 (an estimated 80% of the eligible population). Avon was chosen because it had little outward or inward migration and had a good mix of rural and semi-rural areas, based around the city of Bristol. The initial number of pregnancies enrolled was 14541 (for these at least one questionnaire had been returned or a “Children in Focus” clinic had been attended by 19th July 1999). Of these initial pregnancies, there was a total of 14676 fetuses (resulting in 14062 live births and 13988 children who were alive at 1 year of age). Compared with the whole of Great Britain, ALSPAC mothers were more likely to live in owner-occupied housing, for the household to have a car, to be married, and less likely to be non-white (Fraser et al., 2013).

Comparison with the UK 1991 Census returns from the Avon area indicated that the demography of those who enrolled in ALSPAC was similar to those identified as having a young baby in the rest of the UK (Boyd et al., 2013; Fraser et al., 2013).

Comparison of ALSPAC data on professed religious background in 2001 with the 2001 UK Census and the Bristol district sub-group (the first time religion was measured in the Census) was similar, although the non-religion group was about 10% greater; and the non-Christian groups were slightly less than the rest of the UK.

Data were collected from this cohort throughout childhood and adolescence using sweeps based on the age of the child. Data collection continues using a variety of methods: (a) self-completion questionnaires (including details of the physical and psychological environments, well-being, health and development); (b) hands-on examination of the subjects (including anthropometry, cardiovascular markers, bone health and psychiatric interviews); (c) assays of biological samples (including assays of allergic responses, antibodies indicating recent infections, as well as genetic and epigenetic markers); (d) linkage to statutory data on the individuals (e.g., national education results, health records, cancer registration, deaths); (e) linkage of addresses to measures of geographic exposures (e.g., air pollution, low dose radiation, proximity to green space); (f) schools attended with details of behavior of the child and his/her parents, completed by teachers and headteachers.

At the time of maternal enrollment, and with advice from the ALSPAC Ethics and Law Advisory Committee, it was decided not to enroll the study fathers directly (Birmingham, 2018). A questionnaire was sent for the partner via the mother who could pass this on to him if she would like her partner to be involved. A separate reply-paid envelope was also given for the partner. This methodology meant that the study (deliberately) had no information on whether the mother had invited her partner to take part or not, except on receipt of a completed questionnaire from him. Therefore, reminders could not be sent directly to the partners. In the event, at least one questionnaire was returned by 75% of the partners of women who were enrolled. Please note that the study website contains details of all the data that are available through a fully searchable data dictionary and variable search tool: http://www.bristol.ac.uk/alspac/researchers/our-data/.

Ethical approval for the study was obtained from the ALSPAC Ethics and Law Committee (ALEC) (ALEC IRB00003312) (registered on the Office of Human Research Protections database as UBristol IRB#1) and the three NHS Local Research Ethics Committees (LRECs) that covered the study area (Southmead, Bristol, and Weston and Frenchay). ALEC agreed that consent was implied if questionnaires were returned (Birmingham, 2018). Further detailed information on the ways in which confidentiality of the cohort is maintained and a full list of ethical approvals may be found on the study website: http://www.bristol.ac.uk/alspac/researchers/research-ethics/.

Here we have concentrated on the LOC data collected from questionnaires completed by the mother and/or her partner during pregnancy, and questions concerning religious affiliation and involvement collected during pregnancy and again 6 years later.

### Measures Used

#### Locus of Control

The Adult Nowicki-Strickland Internal External control scale (ANSIE) (Nowicki and Duke, 1974) followed Rotter’s definition in its construction. It has an easier reading level than the Rotter scale, and is significantly correlated with Rotter’s test (Nowicki, 2016) making it appropriate for testing adults from the general population.

A briefer (Anglicized) form of the ANSIE was used in the present study. It contained the 12 items from the original 40 item scale (see Supplementary Appendix 1) which possessed the highest item-total correlations based on the responses in a pilot study carried out on 135 mothers in the US. The scales were completed by each parent at home in mid-pregnancy. Factor analysis of responses from 12471 women confirmed the single factor structure of the scale. Coefficient alpha was 0.78 in this population. The scores ranged from 0 to 12 and were roughly normally distributed with medians of 4 for the mothers (*n* = 12471) and 3 for their partners (*n* = 8645), the higher the score the more external was the LOC. As in our previous publications, external locus of control (ELOC) was defined as above the median while internal locus of control (ILOC) was defined as scores equal to or lower than the median (Golding et al., 2017a, b, 2018; Nowicki et al., 2017; Nowicki et al., 2018).

#### Religious Beliefs

The religious beliefs, attitudes and behavior questions were devised especially for ALSPAC in association with Ursula King (Professor of Theology & Religious Studies at the University of Bristol) in discussion with Jean Golding. The questions were asked separately of both the mother and her partner using self-completion questionnaires during pregnancy and 73 months (6 years) later. The actual wording of the questions is shown in Supplementary Appendix 1 and described in more detail elsewhere (Iles-Caven et al., 2019).

The number of women who answered the antenatal religion/belief questions was 12,351 and those answering the identical questions 5 years later was 8904 (8160 of whom also answered in pregnancy). The study has religion/belief data on 9798 partners antenatally and 4484 at 5 years (4059 on both occasions).

#### Demographic Variables

The demographic variables used to describe the study participants are: (i) the age of the individual at the time their baby was born; (ii) the maximum education level reached, measured in terms of the UK’s national exams or their equivalents; (iii) whether or not the mother was living with the father of the child; (iv) their ethnic origins (grouped together as white/non-white; (v) their place of birth.

### Statistical Analyses

This is a descriptive longitudinal study of a large number of individuals. It is treated as a search for pattern. Comparison is made of internal with externally oriented individuals using a binary classification. Consequently, the data are described and, when appropriate, P values for 2 × n tables are calculated using chi-squared tests. Since this paper is a straightforward description of the association between different aspects of religious beliefs and behaviors and LOC, we did not consider it appropriate to make statistical adjustments for confounders, mediators or moderators. Indeed, it is not easy to distinguish between these. For example, there is considerable evidence that an externally oriented adolescent’s attitudes and behaviors result in lower educational attainment, and thus often in lower levels of occupation (Flouri, 2006). Allowing for such social features would therefore be tantamount to allowing to a certain extent for LOC itself – and thus eliminating the evidence for an association with LOC.

## Results

### Demographic Background

Variation in the demographic backgrounds of the study participants who answered the religiosity questions are shown in Table 1. In general, over 12,000 women and 9000 men were involved. As anticipated the men were slightly more likely to be in the older age group, to be slightly more likely to have higher educational qualifications and to be non-white. The fathers who did not live with the mother were less likely to have been included, presumably because the mother did not pass the questionnaire to her partner. In general, slightly more women had been born in the study area (56.0% of women, 51.3% of men) and more men in the rest of the UK (44.3% of men, 38.9% of women). There were no differences with the sex of the child.

**TABLE 1:** Proportion (n) of study parents who answered the religion questions during pregnancy by various demographic variables.

	Women	Men
**Parental age at birth**		
<25 years	2660(21.6%)	694(10.0%)
25–34 years	8370(68.1%)	4688(67.3%)
35+	1259(10.2%)	1587(22.8%)
**Parental education level**		
<O level (low)	3339(28.7%)	2860(29.5%)
O level (medium)	4075(35.1%)	2193(22.7%)
>O level (high)	4209(36.2%)	4626(47.8%)
**Cohabiting parents**		
Yes	11015(92.0%)	9049(95.5%)
No (including no partner)	956(8.0%)	424(4.5%)
**Sex of child**		
Boy	6411(51.4%)	5089(51.5%)
Girl	6051(48.6%)	4785(48.5%)
**Ethnic background**		
White	11296(97.6%)	8807(97.4%)
Non-white	273(2.4%)	239(2.6%)
**Place of birth of parents**		
Avon	6310(56.0%)	3651(51.3%)
Rest of England (including Channel Islands and Isle of Man)	3827(34.0%)	2779(39.1%)
Wales	316(2.8%)	212(3.0%)
Scotland	167(1.5%)	126(1.8%)
Northern Ireland	67(0.6%)	30(0.4%)
Eire	51(0.5%)	29(0.4%)
Rest of Europe	176(1.6%)	89(1.3%)
Middle East	17(0.2%)	10(0.1%)
Africa	100(0.9%)	77(1.1%)
North, Central and South America	56(0.5%)	19(0.3%)
Caribbean	26(0.2%)	13(0.2%)
Asia	113(1.0%)	50(0.7%)
Australasia	37(0.3%)	27(0.4%)

### Sex Differences in Answers to the Religiosity Questions

The answers given by the study parents to the questions on religion are shown in Table 2. There were strong differences between the responses from the men and women in the study. Almost half of the women (49.9%) stated that they believed in God or some divine being, compared with 36.9% of the men (*P* < 0.001). The proportion who were agnostics (i.e., answered ‘not sure’) were very similar (women 35.3%, men 34.4%), but the proportion of men who declared themselves atheists (answering “no belief”) was almost twice that of women (28.6% vs. 14.9%; *P* < 0.001).

**TABLE 2:** Responses to the questions on religion when asked in pregnancy (*P*-values for comparison of responses of women and men).

	Women	Men	*P*
**Has belief in a divine power**			
Yes	49.9%(6125)	36.9%(3594)	<0.001
Not sure	35.3%(4335)	34.4%(3351)	
No	14.9%(1825)	28.6%(2784)	
**Feel that a divine power has helped**			
Yes	33.9%(4156)	25.3%(2452)	<0.001
Not sure	37.9%(4648)	32.3%(3135)	
No	28.2%(3461)	42.4%(4118)	
**Would appeal to God if in trouble**			
Yes	46.6%(5708)	36.1%(3506)	<0.001
Not sure	31.4%(3846)	27.5%(2671)	
No	22.0%(2700)	36.3%(3526)	
**Duration of faith**			
Life long	81.8%(8854)	79.1%(6621)	<0.001
> 5 years	13.5%(1462)	16.7%(1396)	
≤ 5 years	4.6%(502)	4.3%(358)	
**Frequency of attending a place of worship**			
Weekly	7.4%(886)	6.1%(582)	<0.001
Monthly	6.9%(829)	4.3%(409)	
Annually	29.2%(3508)	26.2%(2500)	
Never	56.5%(6785)	63.4%(6036)	
**Has obtained help from religious leaders**			
Yes	7.7%(892)	6.0%(550)	<0.001
No	92.3%(10676)	94.0%(8659)	
**Has obtained help from members of own religion**			
Yes	9.4%(1086)	7.0%(635)	<0.001
No	90.6%(10408)	93.0%(8484)	
**Has obtained help from members of other religions**			
Yes	2.1%(232)	1.6%(142)	<0.001
No	97.9%(10999)	98.4%(8880)	
**Religious affiliation**			
Church of England	7803(64.3%)	5251(54.8%)	
Roman Catholic	1006(8.3%)	710(7.4%)	
Other Christian (please describe)	950(7.8%)	635(6.6%)	
Judaism	12(0.1%)	8(0.1%)	
Buddhist	27(0.2%)	30(0.3%)	
Sikh	16(0.1%)	18(0.2%)	
Hindu	21(0.2%)	20(0.2%)	
Muslim	55(0.5%)	58(0.6%)	
Rastafarian	5(0.0%)	5(0.1%)	
Other (please describe)	374(3.1%)	374(3.9%)	
“None”	1865(15.4%)	2467(25.8%)	

In line with these results, the men were less likely to have said they had been helped by a divine presence; less likely to appeal to God when in trouble; less likely to attend religious services or to have received help from members of their own or other religious groups. In regard to religious affiliation, whereas proportionately more women than men declared an affiliation with a Christian religion (80.4% women, 68.8% men), slightly more men associated themselves with a non-Christian religion (5.4% men, 4.2% women). As expected from the data on belief described above, the biggest difference between the sexes concerned those declaring no affiliation (15.4% women, 25.8% men). All differences were highly significant (*P* < 0.001).

### Locus of Control and Religious Belief

Table 3 shows the proportion who were internally oriented according to aspects of their beliefs and behaviors. Parents who had a belief in a divine power were more likely to be internally oriented, compared with those who were unsure (i.e., agnostic), who, in turn were more likely to be internal than the non-believers (atheists). The differences between the believers and those who were unsure was much greater than the difference between the unsure and the non-believers.

**TABLE 3:** Proportion (no.) of parents who were internally oriented in pregnancy according to concurrent religious behaviors and beliefs.

	Women	Men
**Has belief in a divine power**		
Yes	60.0% (3673)	58.1% (1661)
Not sure	51.1% (2216)	52.8% (1426)
No	45.1% (823)	49.0% (1098)
	*p* < 0.001	*p* < 0.001
**Feel that a divine power has helped**		
Yes	61.7% (2564)	61.6% (1185)
Not sure	53.5% (2487)	51.1% (1300)
No	47.8% (1654)	51.1% (1694)
	*p* < 0.001	*p* < 0.001
**Would appeal to God if in trouble**		
Yes	60.4% (3448)	58.8% (1637)
Not sure	52.2% (2008)	51.8% (1050)
No	46.1% (1245)	50.0% (1409)
	*p* < 0.001	*p* < 0.001
**Duration of faith**		
Life long	53.7% (4758)	51.9% (2730)
> 5 years	67.9% (993)	69.6% (821)
≤ 5 years	54.6% (274)	51.6% (141)
	*p* < 0.001	*p* < 0.001
**Frequency of attending a place of worship**		
Weekly	73.9% (655)	79.8% (387)
Monthly	67.9% (563)	69.5% (237)
Annually	60.6% (2125)	59.8% (1225)
Never	47.6% (3230)	47.3% (2256)
	*p* < 0.001	*p* < 0.001
**Has obtained help from religious leaders**		
Yes	75.8% (676)	79.4% (375)
No	53.1% (5674)	52.3% (3635)
	*p* < 0.001	*p* < 0.001
**Has obtained help from members of own religion**		
Yes	75.6% (821)	78.9% (422)
No	53.0% (5520)	52.1% (3551)
	*p* < 0.001	*p* < 0.001
**Has obtained help from members of other religions**		
Yes	71.6% (166)	71.1% (86)
No	54.4% (5985)	53.6% (3831)
	*p* < 0.001	*p* < 0.001
**Religious affiliation**		
Christian	54.6% (5324)	53.0% (2784)
Non-Christian	60.6% (309)	62.9% (239)
“None”	54.2% (1010)	54.1% (1106)
	*p* = 0.024	*p* = 0.001

A similar trend in the proportion who were internally oriented between those who were positive, those who were unsure and those who were negative was apparent for those who have, in the past, felt that a divine power had helped, and those who would appeal to God if in trouble. Again, the difference between those who replied positively and the unsure was greater than the difference between the unsure and the negative responses. There were strong differences in orientation between those who obtained help from members of their own or other faiths – those seeking help were more internal.

Individuals who attended a place of worship, even if only annually, were more internal than those who did not attend at all, and there was a trend with frequency of attendance – those who attended weekly being the most internal. When asked about their strategy if in need in the future, those who said that they would appeal to God if they were in trouble were more internal, as were those who were not sure. In contrast, those who stated they would not appeal to God or a divine power were much more likely to be externally oriented (data not shown).

Persons who gave their religious affiliation as other than Christian were more internally orientated than those who were nominally Christian and those with no belief. The same patterns were apparent for both the mothers and the fathers in the study. There was a curious association with the length of time that the individual had had a particular faith – those stating that it was for more than 5 years (but not life-long) were more likely to be internal than those for whom their belief had been apparent for ≤5 years. We investigated this further by analyzing separately those with a firm belief, those with uncertain and those with no belief in a divine being (Supplementary Appendix Tables 1–3). All three groups showed this pattern.

### Locus of Control and Change in Religious Belief

The proportion of men and women who changed their category of belief (i.e., believer; agnostic; atheist) between pregnancy and when their child was aged 6 was similar (26.8% women; 29.3% men) (Table 4). However, there were differences in the proportions who changed according to their LOC orientation in pregnancy: approximately 30% of the women who were external changed during this period of time compared with 25% of those who were internal (*P* < 0.0001). Figures for the men were 32 and 27% respectively (*P* = 0.003).

**TABLE 4:** Numbers changing belief (i.e., belief in God or a divine power) between pregnancy and 6 years.

Mother’s LOC in pregnancy	Women	Men
All	2185/8160 (26.8%)	1189/4059 (29.3%)
External	971/3280 (29.6%)	462/1459 (31.7%)
Internal	1214/4880 (24.9%)	626/1679 (27.2%)
*P*-value	<0.0001	0.003

*P for comparison of External with Internal for women and men separately.*

Tables 5A,B show the actual changes in total numbers of believers reported. In all, the total numbers who stated they were believers 6 years later had fallen by 6.8% (286) of women and 7.7% (120) of men. The overall reductions were somewhat more pronounced among the external than the internal parents: 7.0% (107) vs. 6.6% (179) women; 9.2% (45) vs. 7.8% (75) men. These figures refer to the total numbers – below we describe the movements between groups.

**TABLE 5:** Stability and change in belief in God or a divine power.

Belief in pregnancy	Belief 6 years later				
	Yes	Unsure	No	Total *N* = 100%	*R*
**(A) Mothers**					
*(i) All mothers*					
Yes	80.0%(3386)	17.9%(756)	2.2%(92)	4234	
Unsure	17.8%(501)	64.1%(1807)	18.2%(512)	2820	
No	5.5%(61)	23.8%(263)	70.7%(782)	1106	
					*r* = 0.700
All	48.4%(3948)	34.6%(2826)	17.0%(1386)	8160	
*(ii) External mothers*					
Yes	77.3%(1174)	19.6%(297)	3.2%(48)	1519	
Unsure	17.0%(210)	64.0%(792)	19.1%(236)	1238	
No	5.3%(28)	29.1%(152)	65.6%(343)	523	
					*r* = 0.670
All external	43.0%(1412)	37.8%(1241)	19.1%(627)	3280	
*(iii) Internal mothers*					
Yes	81.5%(2212)	16.9%(459)	1.6%(44)	2715	
Unsure	18.4%(291)	64.2%(1015)	17.4%(276)	1582	
No	5.7%(33)	19.0%(111)	75.3%(439)	583	
					*r* = 0.718
All internal	52.0%(2536)	32.5%(1585)	15.6%(759)	4880	

**(B) Fathers**					
*(i) All fathers*					
Yes	75.0%(1172)	20.3%(317)	4.7%(74)	1563	
Unsure	16.8%(232)	60.0%(826)	23.2%(320)	1381	
No	3.5%(39)	18.8%(207)	77.9%(869)	1115	
All	35.6%(1443)	33.3%(1353)	31.1%(1263)	4059	0.717
(*ii) External fathers*					
Yes	72.0%(352)	21.9%(107)	6.1%(30)	489	
Unsure	15.6%(81)	60.8%(316)	23.7%(123)	520	
No	4.7%(21)	22.2%(100)	73.1%(329)	450	
All	31.1%(454)	35.8%(523)	33.0%(482)	1459	0.673
*(iii) Internal fathers*					
Yes	77.4%(742)	18.8%(180)	3.9%(37)	959	
Unsure	16.6%(126)	60.0%(456)	23.4%(178)	760	
No	2.7%(16)	15.2%(89)	82.1%(481)	586	
All	38.4%(884)	31.5%(725)	30.2%(696)	2305	0.748

*r = correlation coefficient.*

Of those women who were believers in pregnancy, 80% continued to be so, 17.9% had changed to being unsure and only 2.2% had become non-believers. Conversely, of those who had been agnostic 64.1% remained so, 17.8% had become believers, and a further 18.2% had become atheists. Of those who were atheists in pregnancy, 5.5% had become believers and 23.8% agnostic (Table 5A). The results for the men showed that those who believed during pregnancy were slightly less likely to remain as believers compared with the women (75% men, 80% women), somewhat fewer of the agnostics had become believers (17.8% women vs. 16.8% men), and proportionately fewer atheists had become believers (5.5% women vs. 3.5% men). Of those men who were atheists in pregnancy, 77.9% remained so compared with proportionately fewer mothers (70.7%) (Table 5B).

The change in belief was associated with the LOC of the parent – the internal parents who were believers in pregnancy were more likely to stay believers than the external parents who were believers (81.5% internal vs. 77.3% external women; 77.4% vs. 72.0% men). Similarly, the atheists were more likely to remain as atheists when internal compared with the atheists who were external (75.3% internal vs. 65.6% external women; 82.1% vs. 73.1% men) (Tables 5A,B). Assessing the results in an alternative way, the correlation of belief over the 6 years was slightly higher for the internally oriented mothers (*r* = 0.718) than for those who were external (*r* = 0.670). Internally oriented fathers showed a higher correlation than the mothers (*r* = 0.748 v 0.718), but the correlation for the external fathers was similar (*r* = 0.673 vs. 0.670) (Tables 5A,B).

## Discussion

The purposes of the present study were to: (a) evaluate the extent to which religious belief and LOC orientation are associated, and (b) to examine the stability of religious belief and behavior over time in relation to LOC. Using parents in a large longitudinal study we have shown that in this population:

(i) More women than men stated that they believed in God or a divine being; conversely more men than women declared that they had no belief.
(ii) Individuals who were internally oriented were more likely than external individuals to believe, to attend places of worship, to obtain assistance from members of their faith and other faiths.
(iii) In this largely Christian population, those who professed to be affiliated to a non-Christian religion were more internally oriented.
(iv) The internally oriented individuals were more stable in their beliefs than those who were externally oriented over a 6-year period.
(v) Overall there was a reduction over time in this group of parents in the proportion of believers, and an increase in the proportion of non-believers.
(vi) In general, the relationships between LOC and measures of religiosity were similar between men and women.

To our knowledge this is the largest study ever to have assessed the relationships between religiosity and LOC in a population of men and women who were not selected by religion, concurrent illness, educational or occupational group.

As found by others (Fiori et al., 2006; Coursey et al., 2013) women had higher levels of religiosity and were more external than men. As predicted, greater internality was associated with greater religious belief and higher attendance at a place of worship than externality, confirming the findings of Coursey et al. (2013) in their meta-analysis.

The importance of the concept of LOC is illustrated by the large number of studies that have shown associations between internality and outcomes such as higher academic achievement (e.g., Flouri, 2006; Shepherd et al., 2006), greater sporting achievement (e.g., Arnaud et al., 2012) and success in business (e.g., Kormanik and Rocco, 2009; Wu et al., 2015). In contrast, externality is associated with increases in anxiety (Carden et al., 2004), depression (Bjørkløf et al., 2013), negative personality characteristics (Nowicki and Duke, 1974; Wheeler and White, 1991) and psychoses (Harrow et al., 2009; Weintraub et al., 2016).

Rotter conceived LOC as a learned generalized problem-solving expectancy reflecting how much individuals expected their efforts would be necessary in solving academic, social, or, in the present case, existential problems. Considerable research supports the idea that internals deal with practical academic and social problems more effectively than externals (e.g., Nowicki and Duke, 1983, Nowicki and Duke, 2016). However, the broader question of how internals and externals deal with the “problem” of existence has attracted less attention. The results of the present study begin to fill the void concerning information about how internals and externals deal with the problem of intrinsic beliefs. Internals appear to be more stable and intense in their religious beliefs than externals and are more likely to engage in religious activity.

Future research is required to evaluate the association between indicators of extrinsic religiosity and LOC. Extrinsic religiosity is a measure of how individuals use religion for the pursuit of social goals independent of belief, and its relationship with LOC may be reflected differently in the UK and the USA. As part of the UK education system primary schools may be attached to a religion (in the Avon area – the Church of England and Roman Catholic), and may require prospective parents to demonstrate that they attend the church to which the school is affiliated. This might create unknown bias in the research data and needs to be evaluated in future studies.

### Strengths and Limitations

The obvious strengths of this study are: (a) a very large representative set of participants (*n* = over 20,000 men and women) in terms of diversity of social and environmental conditions. (b) The general population was defined geographically and selected without restrictions other than recruitment of pregnant women. (c) The scale used was an appropriate generalized LOC measure for the general population as opposed to Rotter’s which was developed for use by college students. (d) Questions concerning religiosity were asked before the child was born and again 6 years later. (e) Because initial religiosity and LOC questions were asked in pregnancy their responses were not influenced by characteristics of the child to be born.

However, there are a number of limitations and caveats to this study. These include: (i) Not including a measure of extrinsic religiosity; for example, some of the relationships found with LOC, such as attendance at services may be specific to one (extrinsic), whereas religious/spiritual belief may relate more to intrinsic religiosity, rather than both of these measures. (ii) Compared with other areas/countries, there was a relative lack of ethnic diversity of the study population (the population in Avon at the time of testing was mainly white Caucasian and Christian with too few non-white participants for meaningful analysis for specific race or belief systems of participants). (iii) The population studied were adults in the course of pregnancy and 6 years later – the results may not relate to non-pregnant populations of parents, or to individuals who were never pregnant.

One point of criticism is that, instead of analyzing the LOC measure using its continuous data, we have used a dichotomy to divide the externals from the internals. There are several reasons why we have done this. (a) Using the data as continuous can hide problems: for example, it assumes that the differences between any two contiguous integers of score have the same meaning throughout the distribution. This may not be true, especially in a scale measuring psychometric features. (b) Using a dichotomized score makes no assumptions. (c) A division of the population into external and internal makes the results easier for the general population to interpret, and for comparison with other studies. We therefore think that this strategy could be considered an advantage.

A further point of criticism is that there are no sophisticated analyses. This is deliberate. The aim of the study is to describe the data in regard to the research questions. An analysis concerning the pathways from early childhood to belief, and from belief to a lack of belief is complex and awaits further very detailed analysis. The key question in an analysis of LOC and religiosity concerns the causal sequence (if any); in other words, do the individuals who become religious then develop an enhanced internal LOC, or do those with an internal LOC then become religious. These data cannot answer this question, but information collected over 29 years on the offspring of these parents should be able to do so in the future.

## Conclusion

There is a strong association between greater religious belief and a greater internality in both men and women in this population. Since the data were collected simultaneously antenatally, it is impossible to establish causality. However, the longitudinal data show that the non-believer is more likely to become a believer if internally oriented. Future studies involving children of the participants may be able to shed some light on the question as to whether an internal orientation precedes having a religious belief, or vice versa.

## Data Availability Statement

The datasets generated for this study will not be made publicly available: the ALSPAC study website contains details of all the data that are available through a fully searchable data dictionary and variable search tool: http://www.bristol.ac.uk/alspac/researchers/our-data/. Applications to use data should be made to ALSPAC.

## Ethics Statement

Ethical approval for the study was obtained from the ALSPAC Ethics and Law Committee and the three Local Research Ethics Committees (LRECs) that covered the study area. The ALSPAC Ethics and Law Advisory Committee agreed that consent was implied if questionnaires were returned. Written informed consent for participation was not required for this study in accordance with the national legislation and the institutional requirements.

## Author Contributions

JG planned and carried out the analyses with SG and GE. YI-C and JG wrote the first draft. All authors were involved in editing, checking, and rewriting the manuscript.

## Conflict of Interest

The authors declare that the research was conducted in the absence of any commercial or financial relationships that could be construed as a potential conflict of interest.
