# Supplementary material for: The Relationship Between Locus of Control and Religious Behavior and Beliefs in a Large Population of Parents: An Observational Study
Source: Front Psychol. 2020 Jun 25;11:1462. doi: 10.3389/fpsyg.2020.01462 (PMC7330129; doi:10.3389/fpsyg.2020.01462)
Supplement: Supplementary file 1 [file Data_Sheet_1.PDF]

Appendix 1

Questions asked of the mother and father (antenatally and 6 years post-partum)

Religious belief

Do you believe in God or in some divine power? Yes/Not sure/No

Do you feel that God (or some divine power) has helped you at any time? Yes/Not sure/No

Would you appeal to God for help if you were in trouble? Yes/Not sure/No

What sort of religious faith would you say you had? (tick only one)
None/Church of England/Roman Catholic/Jehovah's Witness/Christian Science/Mormon/ Other Christian (please describe)/Jewish/Buddhist/Sikh/Hindu/Muslim/Rastafarian/ Other (please describe)

How long have you had this particular faith?
All my life/More than 5 years/3-5 years/1-2 years/Less than a year

Do you go to a place of worship?
Yes, at least once a week/Yes, at least once a month/Yes, at least once a year/Not at all

Do you obtain help and support from leaders or other members of groups? Yes/No
From Leaders of your religious group (e.g. priests, rabbis, imams)? Yes/No
From other members of your religious group? Yes/No
From members of other religious groups (please describe)? Yes/No

Locus of control scale (ANSIE)

1.Did getting good marks at school mean a great deal to you? Yes/No

2.Are you often blamed for things that just aren't your fault? Yes/No

3.Do you feel that most of the time it doesn't pay to try hard because things never turn out right anyway? Yes/No

4.Do you feel that if things start out well in the morning that it's going to be a good day no matter what you do? Yes/No

5.Do you believe that whether or not people like you depends on how you act? Yes/No

6.Do you believe that when bad things are going to happen they are just going to happen no matter what you try to do to stop them? Yes/No

7.Do you feel that when good things happen they happen because of hard work? Yes/No

8.Do you feel that when someone doesn't like you there's little you can do about it? Yes/No

9.Did you usually feel that it was almost useless to try in school because most other children were cleverer than you? Yes/No

10.Are you the kind of person who believes that planning ahead makes things turn out better? Yes/No

11.Most of the time, do you feel that you have little to say about what your family decides to do? Yes/No

12.Do you think it's better to be clever than to be lucky? Yes/No

N.B. For creating the LOC score, questions 2, 3, 4, 6, 8, 9 and 11 were coded as yes = 0, no = 1; the remaining questions were coded as yes = 1, no = 0. The responses were then summed.

Appendix Table 1. Parents who believed in God or a divine power: proportion who were internally oriented

	**WOMEN**	**MEN**
Feel that a divine power has helped		
Yes	62.1% (2412)	62.5% (1097)
Not sure	58.1% (1072)	52.2% (431)
No	47.9% (182)	47.9% (128)
	p<0.001	p<0.001
Would appeal to God if in trouble		
Yes	62.0% (2916)	60.7% (1248)
Not sure	55.4% (631)	53.4% (284)
No	44.7% (117)	47.3% (122)
	p<0.001	p<0.001
Duration of faith		
Life long	58.3% (2808)	54.6% (1131)
>5 years	69.3% (605)	72.8% (397)
≤5 years	59.0% (184)	57.5% (103)
	p<0.001	p<0.001
Frequency of attending a place of worship		
Weekly	74.4% (647)	78.0% (379)
Monthly	68.8% (503)	69.6% (192)
Annually	61.5% (1401)	58.7% (588)
Never	49.5% (1076)	45.1% (493)
	p<0.001	p<0.001
Has obtained help from religious leaders		
Yes	76.1% (655)	79.3% (356)
No	57.1% (2827)	54.3% (1267)
	p<0.001	p<0.001
Has obtained help from members of own religion		
Yes	76.4% (795)	78.5% (398)
No	56.8% (2695)	53.8% (1199)
	p<0.001	p<0.001
Has obtained help from members of other religions		
Yes	71.9% (151)	74.7% (74)
No	59.3% (3157)	57.4% (1470)
	p<0.001	p=0.001
Religious affiliation		
Christian	60.1% (3370)	58.1% (1452)
Non-Christian	59.5% (207)	61.8% (141)
"None"	57.4% (62)	51.9% (54)
	p=0.830	p=0.230

Appendix Table 2. Parents who were unsure of their belief in God or a divine power: proportion who were internally oriented

	WOMEN	MEN
Feel that a divine power has helped		
Yes	56.0% (145)	53.2% (84)
Not sure	50.8% (1387)	50.9% (838)
No	51.0% (680)	56.1% (499)
	p=0.276	p=0.044
Would appeal to God if in trouble		
Yes	53.4% (520)	54.1% (359)
Not sure	51.1% (1287)	50.8% (724)
No	48.6% (402)	55.7% (336)
	p=0.117	p=0.093
Duration of faith		
Life long	50.5% (1649)	52.4% (1096)
>5 years	66.3% (187)	62.5% (155)
≤5 years	44.1% (60)	46.2% (18)
	p<0.001	p=0.007
Frequency of attending a place of worship		
Weekly	46.2% (6)	87.5% (7)
Monthly	60.6% (57)	70.4% (38)
Annually	59.3% (666)	60.7% (506)
Never	48.0% (1436)	48.1% (845)
	p<0.001	p<0.001
Has obtained help from religious leaders		
Yes	66.7% (18)	78.9% (15)
No	51.4% (2111)	52.7% (1362)
	p=0.114	p=0.022
Has obtained help from members of own religion		
Yes	55.8% (24)	84.0% (21)
No	51.5% (2093)	52.7% (1350)
	p=0.575	p=0.002
Has obtained help from members of other religions		
Yes	70.6% (12)	33.3% (<5)
No	51.4% (2095)	53.0% (1361)
	p=0.115	p=0.174
Religious affiliation		
Christian	49.8% (1758)	51.9% (1109)
Non-Christian	60.2% (71)	63.3% (57)
“None”	57.6% (349)	55.5% (239)
	p<0.001	p=0.054

Appendix Table 3. Parents who did not believe in God or a divine power: proportion who were internally oriented

	WOMEN	MEN
Feel that a divine power has helped		
Yes	50.0% (<5)	40.0% (<5)
Not sure	39.4% (26)	40.0% (28)
No	45.4% (792)	49.3% (1062)
	p=0.621	p=0.260
Would appeal to God if in trouble		
Yes	30.4% (7)	47.5% (28)
Not sure	47.8% (89)	53.7% (117)
No	45.1% (724)	48.6% (949)
	p=0.277	p=0.349
Duration of faith		
Life long	38.6% (290)	45.3% (492)
>5 years	65.2% (199)	69.5% (267)
≤5 years	54.7% (29)	36.4% (20)
	p<0.001	p<0.001
Frequency of attending a place of worship		
Weekly	50.0% (<5)	– (0)
Monthly	66.7% (<5)	60.0% (6)
Annually	54.2% (52)	60.8% (127)
Never	44.3% (710)	47.7% (909)
	p=0.246	p=0.002
Has obtained help from religious leaders		
Yes	75.0% (<5)	100.0% (<5)
No	45.5% (725)	49.2% (992)
	p=0.236	p=0.079
Has obtained help from members of own religion		
Yes	50.0% (<5)	100.0% (<5)
No	45.7% (722)	49.3% (988)
	p=0.902	p=0.152
Has obtained help from members of other religions		
Yes	50.0% (<5)	80.0% (8)
No	45.8% (723)	49.3% (1361)
	p=0.904	p=0.052
Religious affiliation		
Christian	30.3% (180)	35.6% (215)
Non-Christian	73.2% (30)	65.6% (40)
“None”	52.0% (597)	53.8% (808)
	p<0.001	p<0.001
